# Tetraspanin CD53: an overlooked regulator of immune cell function

**DOI:** 10.1007/s00430-020-00677-z

**Published:** 2020-05-21

**Authors:** V. E. Dunlock

**Affiliations:** Department of Tumor Immunology, Radboud Institute for Molecular Life Sciences, Radboud University Medical Center, Nijmegen, The Netherlands

**Keywords:** Tetraspanins, Tetraspanin enriched microdomains, CD53, Membrane organization, Immune cell signaling, Immune cell adhesion

## Abstract

Tetraspanins are membrane organizing proteins that play a role in organizing the cell surface through the formation of subcellular domains consisting of tetraspanins and their partner proteins. These complexes are referred to as tetraspanin enriched microdomains (TEMs) or the tetraspanin web. The formation of TEMs allows for the regulation of a variety of cellular processes such as adhesion, migration, signaling, and cell fusion. Tetraspanin CD53 is a member of the tetraspanin superfamily expressed exclusively within the immune compartment. Amongst others, B cells, CD4^+^ T cells, CD8^+^ T cells, dendritic cells, macrophages, and natural killer cells have all been found to express high levels of this protein on their surface. Almost three decades ago it was reported that patients who lacked CD53 suffered from an increased susceptibility to pathogens resulting in the clinical manifestation of recurrent viral, bacterial, and fungal infections. This clearly suggests a vital and non-redundant role for CD53 in immune function. Yet, despite this striking finding, the specific functional roles of CD53 within the immune system have remained elusive. This review aims to provide a concise overview of the published literature concerning CD53 and reflect on the underappreciated role of this protein in immune cell regulation and function.

## Introduction: tetraspanins in the immune system

There are 33 known tetraspanin family members expressed in human cells. Tetraspanins are integral membrane proteins containing four transmembrane domains, two extracellular domains, and two short intracellular tails [1]. Tetraspanins are approximately 20–25 kDa in size and defined by the presence of four, six or eight conserved cysteine domains found within the second extracellular loop (EC2) [2]. Tetraspanins are membrane organizing proteins that form microdomains by exploiting their ability to interact in cis with each other and with partner proteins located either on the cell surface or on intracellular membranes. Specific α-helices located within the EC2 exhibit high variability allowing the different tetraspanins to interact specifically with certain partner proteins. Through these interactions they are able to organize proteins into membrane microdomains referred to as tetraspanin enriched microdomains (TEMs) or the tetraspanin web [3]. TEMs bring together functionally related proteins to regulate different cellular processes, including adhesion, migration, signaling, activation, and cell fusion [4–7]. Well-characterized tetraspanin partners include integrins, co-stimulatory molecules, and protein kinase C (PKC) [6, 8, 9]. Though the majority of tetraspanins have a ubiquitous expression pattern, there are two tetraspanins which are known to be restricted to the immune compartment, namely, CD37 and CD53. CD37 is predominantly expressed on B cells with lower expression found in other immune cells [10]. CD53, on the other hand, was found to have a high and comparable expression over different immune cell types, possibly indicating a more general function within the immune system. Furthermore, CD37 has been found to possess ITIM-like and ITAM-like domains which are proposed to allow for direct signaling by CD37, while this is not the case for CD53 [11]. In terms of partner protein interactions, CD37 and CD53 have a few common partners such as β1 integrins and major histocompatibility complex proteins (MHC), whereas others are tetraspanin-specific, for example the interaction between PKC and CD53 or between suppressor of cytokine signaling 3 (SOCS3) and CD37 [12–14].

CD37 has been extensively studied and its loss has been linked to defects in numerous cellular and humoral immune functions such as migration, adhesion, proliferation, and antibody production [15–18]. The absence of CD37 results in spontaneous development of B cell lymphoma in mice and has been shown to correlate with worse progression-free and overall survival in lymphoma patients [13, 19]. In addition, multiple tetraspanins including CD37 have been shown to play a role in T cell activation, co-stimulation, proliferation, and immunological synapse formation [20, 21].

In contrast, CD53, also known as OX44 or TSPAN25, has been comparatively understudied despite strong evidence that this tetraspanin has important and non-redundant functions within the immune system [22–25]. CD53 is expressed highly on B cells and myeloid cells, though T cells also express it at significant levels [10, 26]. While there are many unanswered questions with respect to CD53, there are some functions specifically related to cell adhesion and signaling that have been ascribed to this protein, particularly in B and T cells. An overview of the proposed interaction partners of CD53 is shown in Table 1.

**Table 1:** An overview of potential CD53 partner proteins on immune cell subsets and the functional implications of these interactions

Potential partner	Immune cell type	Functional consequences	Refs.
LFA-1 (αLβ2)	B cells, T cells and NK cells	(Homotypic) adhesion	[22, 27]
VLA-4 (α4β1)	B and T cells	Unknown	[28]
L-selectin	B and T cells	Disrupted lymphocyte recirculation and impaired immunity	[25]
PKCs	B cells, T cells and macrophages	PKC translocation and activation	[24, 29–31]
Unknown partner with Akt as a downstream effector	B and T cells	Cell survival	[32]
Unknown partner with JNK as a downstream effector	B and T cells	Activation of Jun dependent transcription	[33]
Unidentified Tyr. phosphatase	Lymph node and thymoma cells	Unclear, possible role in Lck dephosphorylation	[34]
MHCI	B cells	Unknown	[14]
MHCII	B cells	Unknown	[14, 35]
CD2	T and NK cells	Possible role in signaling and cell activation	[36]
IL-7R	B cells	Supports survival and differentiation of B cells	[37]
γ-glutamyl transpeptidase (GGT)	B cells	Functional significance unknown	[38]

The aim of this review is to present a framework for the unique function of CD53 within the immune system based on the published literature. I will discuss the regulatory role of CD53 in different cellular processes with a focus on immune cell adhesion and signaling.

## The role of CD53 in immune cell adhesion and migration

The ability of cells to adhere to one another and to the extracellular matrix (ECM) is important for maintaining the architecture and integrity of tissues and enabling cells to create the forces required for movement. Cell adhesion depends on cell adhesion molecules (CAMs) expressed on the cell surface, which include integrins, selectins, and cadherins. Cell adhesion plays an especially important role in the immune system, where adhesion molecules are used to stabilize immunological synapses between immune cells and facilitate their migration to sites of inflammation or infection [39, 40].

Many tetraspanins have been proposed to interact with CAMs, of which integrins in particular have been found to be key interaction partners [8, 41]. Integrins are heterodimeric adhesion molecules composed of an alpha and a beta subunit. They are involved primarily in cell–cell interactions and interactions between cells and the extracellular matrix (ECM). Tetraspanins have been reported to regulate integrin function through modulation of integrin signaling, by changing the distribution of integrins on the cell surface or by directing the trafficking of integrins [15, 42] For example, in T cells tetraspanins, CD9 and CD151 were found to accumulate at the immunological synapse. In the absence of these two tetraspanins integrin VLA-4 (α4β1) failed to accumulate at the cell–cell contact site which resulted in reduced T cell activation [20].

CD53 has also been linked to integrins and implicated in the regulation of adhesion of multiple immune cell types. Traditionally, the role of CD53 in adhesion has been studied using antibody ligation of CD53. For example, Todros-Dawda et al. showed that ligation of CD53 induced activation of the β2 integrin LFA-1 (αLβ2) in natural killer (NK) cells resulting in homotypic adhesion [22]. Furthermore, they proposed that CD53 ligation shifts NK cells towards a more proliferative phenotype, based on observed reductions in the degranulation responses of NK cells towards tumor cells, and impaired cytokine production.

The interaction between CD53 and LFA-1 is not restricted to NK cells as similar results have also been obtained in other immune cell types. In B and T cell lines the ligation of CD53, either via whole antibodies or via Fab’ fragments, also resulted in homotypic cell adhesion, which could be prevented through pre-treatment with antibodies against LFA-1 or its ligand ICAM-1 [27]. Based on these findings, the authors proposed that ligation of CD53 may affect cell activation resulting in increased cell adhesion which facilitates leukocyte adherence, extravasation, and aggregation to endothelia at sites of inflammation.

These findings were contradicted by the work of Lazo et al. who found that blocking of LFA-1 did not relieve homotypic adhesion of rat B cells induced by CD53 ligation [31]. Suggesting that this process may involve other adhesion molecules instead of, or in addition to, LFA-1. Supporting the involvement of other adhesion molecules, the authors found that the homotypic B cell adhesion could be prevented through addition of the chelating agents EDTA and EGTA, which remove the divalent cations required for integrin function. An alternative integrin to LFA-1 that may be involved is VLA-4, which has also been linked to CD53 through co-immunoprecipitation experiments, though the functional consequences of this interaction have remained unstudied [28]. Lazo et al. did not comment on the possible involvement of other integrins. Instead the authors suggested based on the application of inhibitors that signaling through tyrosine kinases and PKC might be responsible for the increased adhesive capacity of these cells. Moreover, the homotypic adhesion of these rat B cells was also completely abolished by inhibition of phosphoinositide 3-OH kinase (PI3K). The authors further supported this hypothesis by showing that de novo protein synthesis was a requisite for the homotypic adhesion induced by CD53 ligation, suggesting the involvement of intracellular signaling pathways.

While the abovementioned studies have made a good case for the involvement of CD53 in immune cell adhesion, they all suffered from the same shortcoming, namely, the exclusive use of antibodies to study CD53 function. While this method has been useful to identify potential partners of CD53, the results should be interpreted with caution as Fc receptor cross-linking may also play a role in the observed experimental outcomes. Modulation of the expression on CD53 through knockdowns, knockouts or overexpression are all viable alternatives to this method, as is illustrated by the following study.

Using an elegant *Cd53*^*−/−*^ mouse model, Demaria et al. have shown that the homing of *Cd53*^*−/−*^ lymphocytes to the lymph node is defective, with B cells more severely affected than T cells [25]. This was due to a severe decrease in the expression of the lymph node homing receptor L-selectin in *Cd53*^*−/−*^ B cells. The impaired L-selectin expression in the absence of CD53 was confirmed in two human *CD53*^*−/−*^ B cell lines. Further experiments revealed that the phenotype could be partially rescued by the application of a metalloprotease inhibitor suggesting that metalloproteases are partly responsible for the decrease in L-selectin expression when CD53 is absent. This was confirmed in experiments showing that CD53 inhibits the L-selectin shedding via ADAM17-dependent and -independent mechanisms. Finally, the authors showed that this defect in lymph node homing caused by the absence of CD53 resulted in a delayed adaptive immune response upon in vivo challenge with model antigens, illustrating the importance of this tetraspanin in the adaptive immune response.

Taken together the work discussed here illustrates the vital and varied role CD53 plays in regulating immune cell adhesion and migration, controlling this process through multiple distinctive adhesion molecules in different cell types. In addition, the work of Demaria et al. specifically, highlights the negative consequences associated with the absence of CD53 which result in pronounced immune dysfunction associated with an adhesion-related migration defect.

**Fig. 1:**
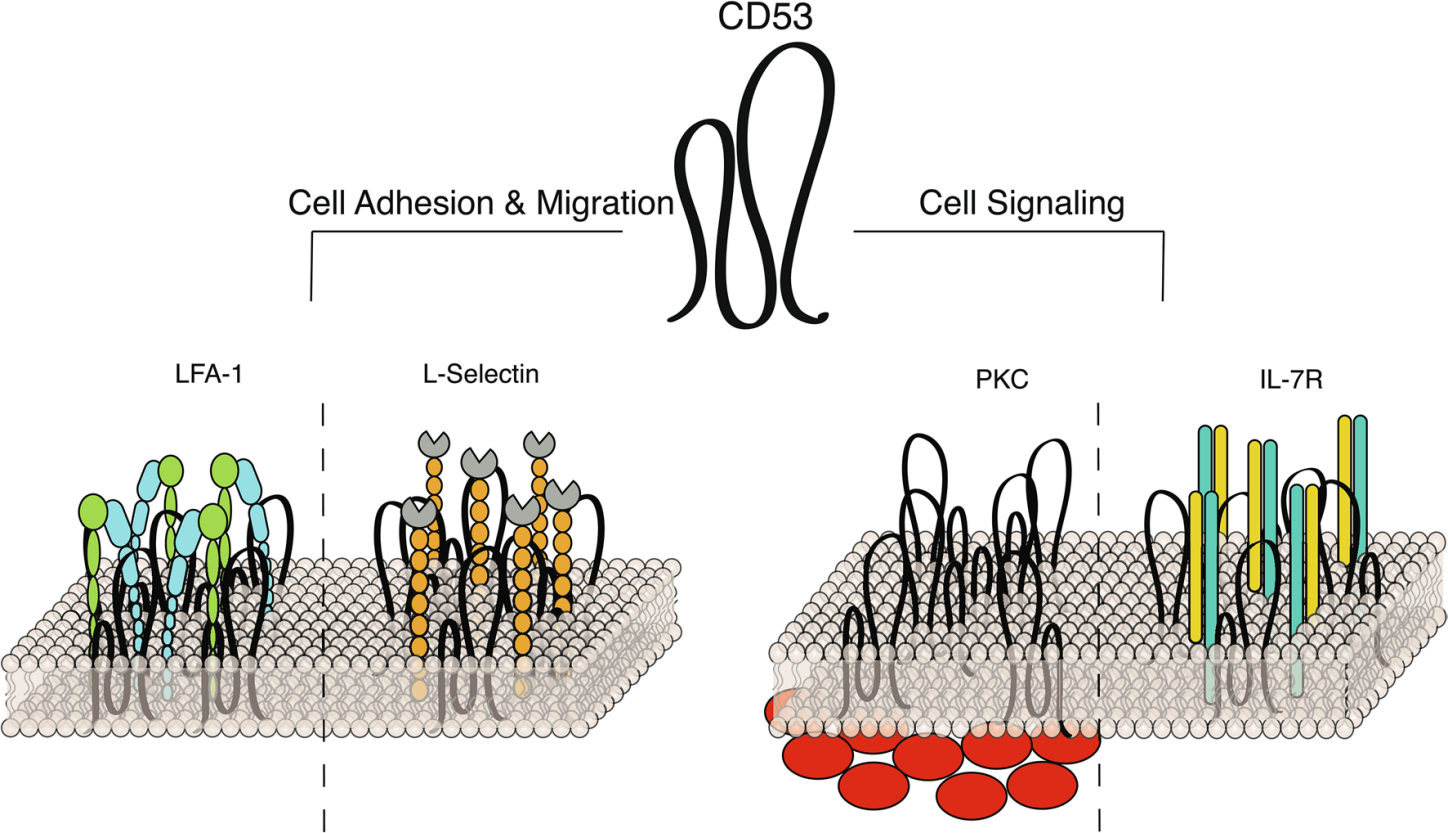
Tetraspanin CD53 interacts with both membrane and cytosolic proteins to regulate immune cell function. A schematic overview of selected interactions between tetraspanin CD53 and adhesion molecules (left) and signaling molecules (right) in immune cells. Details can be found in the main text

An overview of the most prominent interactions discussed in this section can be found in Fig. 1.

## CD53 as a regulator of immune cell signaling

A healthy immune system depends on the intricate and reciprocal relationship that exists between immune cells and their environment. Central to this cellular interplay is the process of signaling, which involves receiving, transducing and responding to signals provided by neighboring cells or by the external surroundings. As the boundary between the intracellular and extracellular environment, the plasma membrane is vital to this process, providing a dynamic platform through which receptors activate signaling pathways.

Though signaling is a ubiquitous cell function, it has a particularly important role within the immune system which is dependent on signaling to recognize and eliminate pathogens and transformed cells. Tetraspanins help to support this function by forming TEMs that can function as platforms to support intense signaling activity. Different interactions between tetraspanins and signaling proteins have been reported, including both kinases and phosphatases [43].

CD53 has been found to interact with numerous membrane proteins involved in signaling. For example, CD2, which is expressed on the surface of NK and T cells, plays a role in cell adhesion and functions as a co-stimulatory molecule [44]. CD2 was identified as a partner of CD53 based on a screening of antibodies found to activate the phosphatidylinositol signaling pathway in a rat leukemia cell line with NK activity (RNK-16) [36]. Multiple antibodies identified in this screening were targeted to CD53, and were shown to elicit tyrosine phosphorylation, generate inositol phosphates and increase mobilization of calcium to the cytoplasm. Further investigation revealed that CD53 co-immunoprecipitated with CD2 in both NK and primary rat T lymphocytes and that stimulation via CD53 augmented TCR-mediated proliferation. Whether CD53 plays a role in the in vivo physiological activation of NK and T cells and whether this involves association with CD2 still remains unknown.

More recently a role for CD53 has been recognized as a regulator of B cell development via interleukin 7 receptor (IL-7R) signaling [37]. The authors identified a direct and specific interaction between CD53 and IL-7R through co-immunoprecipitation and proximity ligation assays. Subsequent experiments showed that B cells of mice lacking CD53 had a reduced surface expression of IL-7R accompanied by a significant decrease in bone marrow, splenic, lymphatic and peripheral B cells. These B cells also exhibited diminished PI3K and JAK/STAT signaling in the pro-B and pre-B cell developmental stage. The decrease in IL-7R expression is of interest because signaling via this receptor is required for early B cell survival and transition from pro-B to pre-B cell. In line with this, the authors reported increased cell death in developing *Cd53*^*−/−*^ B cells and an associated reduction in pre-B and immature B cell populations. Not only does this study serve to further cement the role of CD53 in signaling, it also supports the premise that the immune system is reliant on CD53 in important and non-redundant ways.

Finally, numerous tetraspanins, including CD53, have been reported to associate with MHC class I (MHC-I) and class II (MHC-II) proteins [14]. CD53 was found to be part of a large complex on the surface of B cells which consisted of multiple tetraspanins, CD20 and MHC-I and -II. The authors speculated that these supramolecular complexes might play a role in signaling through MHC molecules and in antigen presentation to T cells, though they provided no evidence to support either claim. This proposed association is supported by findings from our own group using both immunoprecipitation and super-resolution microscopy which showed that CD53 could be immunoprecipitated with MHC-II and that CD53 was closer in proximity to MHC-II on the cell surface than other tetraspanins, indicating an association between these proteins [35]. In general, research into the relationship between tetraspanins and MHC molecules remains somewhat controversial due to conflicting results and the use of an antibody to identify tetraspanin-MHC interactions which later proved to be unspecific [45, 46]. Further research is required to concretely prove whether CD53 truly interacts with MHC molecules and what the functional consequences of this interaction may be.

Beyond membrane protein interactions, tetraspanins also have the ability to interact directly with intracellular signaling molecules, forming membrane hotspots for intense signaling activity. The foremost example of this is the interaction between tetraspanins and protein kinase C (PKC), which is especially relevant to tetraspanin CD53. In jurkat T cells PKCα has been found to co-immunoprecipitate reciprocally with numerous tetraspanins [29]. The authors observed that several tetraspanins, including CD53, created complexes that could link PKCs and integrins to each other to facilitate the PKC-dependent phosphorylation of integrins. This was supported by the finding that only integrins capable of interacting strongly with tetraspanins were found to interact with PKC. Further strengthening these finding is the work of Boscá et al. who showed that in rat macrophages the ligation of CD53 resulted in the mobilization of intracellular calcium and subsequent activation of PKC [30]. Similar CD53-mediated calcium fluxes have been observed in B cells, monocytes and granulocytes further substantiating this observation [47]. More recently our group has confirmed the existence of a direct interaction between CD53 and PKC in living immune cells [24]. In B cells we have shown that in the absence of CD53 the recruitment of PKCβ to the plasma membrane is impaired upon B cell receptor stimulation. This has implications for the activation of B cells, with CD53-negative primary B cells exhibiting impaired phosphorylation of PKC substrates. We were able to show that this is a result of a direct interaction between CD53 and PKCβ, as the deletion of a portion of the N-terminus of CD53 abrogates this interaction.

PKC is not the only kinase CD53 has been linked to. In B and T lymphoma cell lines the use of antibodies to ligate CD53 resulted in increased protein kinase B (Akt) phosphorylation which reduced the number of cells entering apoptosis upon serum deprivation [32]. Unfortunately, the intermediary between CD53 and the Akt pathway, whether this is a membrane or cytosolic protein, remains unknown. The authors proposed that this is a survival mechanism employed by tumor cells in poorly vascularized areas where nutrients and oxygen are scarce. The role of Akt in survival signaling is linked to the upstream kinase PI3K, which has been implicated in signaling related to CD53 as discussed above [31].

Like Akt, c-Jun N-terminal kinase (JNK) has also been identified as an effector protein functioning downstream of CD53 [33]. The antibody ligation of CD53 in both B and T cell lines was found to result in a fourfold increase in JNK activation which was transient and peaked at 3–5 min. In addition, the authors showed similar JNK activation in cells that normally do not express CD53 (renal cells, fibroblast) upon overexpression of CD53, suggesting that the role of CD53 in JNK activation is cell type independent. As with Akt, the mediator connecting CD53 to the JNK pathway is yet to be identified.

Kinases are not the only signaling molecules associated with CD53, almost three decades ago Carmo et al. provided initial evidence of an interaction between CD53 and an unidentified tyrosine phosphatase in lymph node cells and in a thymoma cell line [34]. This was based on immunoprecipitation experiments in which CD53-containing immune complexes were pulled down followed by kinase assays in the presence or absence of phosphatase inhibitors. These studies revealed that CD53 was associated with a tyrosine phosphatase capable of dephosphorylating Lck. The authors went on to present evidence that this tyrosine phosphatase was likely not CD45, but no further indication was provided as to the specific identity of this tyrosine phosphatase. Despite remaining unidentified, these results demonstrate that CD53 has a versatile function regulating both kinases and phosphatases and thereby contributing to the overall equilibrium of phosphorylation driving immune cell activation.

An overview of the most important interactions discussed in this section can be found in Fig. 1.

## Outlook

Despite the fact that we have known for over 25 years that CD53 plays an important and non-redundant role in the immune system, it is only recently that we have begun to unravel the actual function of this tetraspanin [23]. Here, I have summarized the relevant literature regarding CD53 and its function within the immune system as a regulator of immune cell adhesion and signaling. Based on the data discussed in this review, I posit that CD53 has proven itself to be a versatile and important molecule in immune function worthy of further investigation.

The literature examined in this review gives rise to a number of unresolved subjects and questions I will discuss here. For example, CD53 is expressed on all cells of the lymphoid-myeloid lineage, yet the majority of research has been focused exclusively on B and T cell lines (Table 1). It would be of great benefit to explore the role of CD53 in other immune cells (such as antigen-presenting cells) and to further extend findings to primary cells and in vivo models.

Furthermore, the greater majority of research described here has relied on the use of antibody ligation to study CD53 function. Though this has no doubt been incredibly valuable in the past, we would be remiss not to take more advantage of the opportunities presented by novel techniques like CRISPR/Cas9 and the tetraspanin knockout mice established in the recent past to investigate CD53 function further. These tools allow for much more flexibility than the traditional antibody-based method and would permit us to begin designing studies to build on the fundamental knowledge we have gained in the last years.

In line with this, a strong case has been made for the interaction between CD53 and PKCs, which presents a unique opportunity to target this ubiquitous signaling molecule through an accessible surface protein. For many years, PKC inhibitors have been a target for intense research as an anti-cancer therapy [48]. These efforts have been hampered by the broad expression pattern of PKCs resulting in undesired adverse effects [49–51]. Based on this, it would be worthwhile to investigate the possibility of targeting PKC via CD53 in hematological malignancies, which provides not only an easily accessible membrane target but also an increased degree of specificity. Moreover, research has shown that the expression of CD53 is very high in radioresistant tumor cells, making this an even more attractive prospect as it may provide a new option in difficult to treat malignancies [52].

Beyond the matters raised in the literature discussed here, there are also many fundamental open questions related to CD53 which have not been addressed. For example, the exact composition of CD53 TEMs is still largely undefined. Which other partner proteins and lipids are contained by these microdomains and how do these contribute to CD53 function? Specifically, in the case of signaling there are numerous downstream pathways that have been linked to CD53, but the partner molecules mediating these effects have remained undefined.

Likewise, it would be interesting to further study what happens during activation of immune cells when CD53 is absent or impaired in its function. This is particularly interesting because cell activation is dependent on both the adhesive/migratory capacity of cells as well as their ability to signal, both of which have been linked to CD53 function. How CD53 would go about offsetting these functions in such a setting is therefore very interesting, particularly from the point of view of spatiotemporal dynamics. Finally, since CD53 has been proposed to interact with both kinases and phosphatases, it would be interesting to investigate how CD53 contributes to balancing signaling responses and whether this is dependent on factors such as timing, signal intensity, etc.

In conclusion, it is time to embrace a novel view of the immune system which recognizes the importance of membrane organizing proteins like CD53 and the central roles they can play in immunological processes. As a consequence, questions concerning how these organizing proteins function become increasingly important to our understanding of pathologies and the development of novel therapeutic strategies.
